# On the Global Practical Exponential Stability of *h*-Manifolds for Impulsive Reaction–Diffusion Cohen–Grossberg Neural Networks with Time-Varying Delays

**DOI:** 10.3390/e27020188

**Published:** 2025-02-12

**Authors:** Gani Stamov, Trayan Stamov, Ivanka Stamova, Cvetelina Spirova

**Affiliations:** 1 Department of Mathematics, University of Texas at San Antonio, San Antonio, TX 78249, USA; gani.stamov@utsa.edu; 2 Department of Engineering Design, Technical University of Sofia, 1000 Sofia, Bulgaria; tstamov@tu-sofia.bg; 3 Department of Mathematics, Technical University of Sofia, 8800 Sliven, Bulgaria; cvetelina2802@abv.bg

**Keywords:** Cohen–Grossberg neural networks, reaction–diffusion terms, impulses, practical stability, h-manifolds

## Abstract

In this paper, we focus on *h*-manifolds related to impulsive reaction–diffusion Cohen–Grossberg neural networks with time-varying delays. By constructing a new Lyapunov-type function and a comparison principle, sufficient conditions that guarantee the global practical exponential stability of specific states are established. The states of interest are determined by the so-called *h*-manifolds, i.e., manifolds defined by a specific function *h*, which is essential for various applied problems in imposing constraints on their dynamics. The established criteria are less restrictive for the variable domain and diffusion coefficients. The effect of some uncertain parameters on the stability behavior is also considered and a robust practical stability analysis is proposed. In addition, the obtained *h*-manifolds’ practical stability results are applied to a bidirectional associative memory (BAM) neural network model with impulsive perturbations and time-varying delays. Appropriate examples are discussed.

## 1. Introduction

Recently, the study of the qualitative behavior of Cohen–Grossberg neural networks has attracted wide attention due to implementing of its ample applications. In fact, this important class of neural networks introduced in 1983 [1] has been widely used as mathematical models in engineering, neuroscience, biology and medicine. As such, the stability, periodicity, almost periodicity and other qualitative properties of Cohen–Grossberg neural networks have been intensively investigated [2,3,4]. Note that this class of neural network systems is quite general and includes several well-known neural networks, such as Hopfield neural networks, Lotka–Volterra systems, Kolmogorov systems and cellular neural networks. It is also worth mentioning that some entropy phenomena are related to efficient neural network training, and entropy is applied to measure the complexity in neural network architectures [5,6].

The effects of time delays on the performance of neural network models is very well known; hence, great progress in the study of delayed Cohen–Grossberg neural network models has been made. Models with constant delays, as well as more general cases involving time-varying delays, have been investigated [7,8,9,10].

The fact that reaction–diffusion effects can disrupt the high performance of neural networks has greatly enhanced the study of the dynamic behavior of neural networks with reaction–diffusion conditions [11,12,13]. The type of Cohen–Grossberg neural networks with reaction–diffusion terms has also been intensively investigated [14,15,16,17]. Indeed, reaction–diffusion neural networks constitute a suitable framework to study dynamics in neural network processes that not only dependent on the evolution time but also dependent on space.

Due to the development of impulsive control strategies, impulsive neural networks have drawn more attention recently, including impulsive Cohen–Grossberg neural networks [18,19,20,21,22]. Such models include impulsive conditions that describe the behavior of states at some discrete instances. In the study of their properties, the theories and apparatus of impulsive differential equations and impulsive control dynamical systems are used [23,24,25,26]. In fact, through suitable short-term perturbations, researchers can control some qualitative properties of neural network models, such as stability, stabilization, synchronization, periodicity, almost periodicity and some others. Hence, the investigations on impulsive Cohen–Grossberg reaction–diffusion neural networks have also increased rapidly. For example, in [27], the existence, uniqueness and global exponential stability of the equilibrium state for an impulsive Cohen–Grossberg neural network with distributed delays and reaction–diffusion terms have been investigated. The uncertain case was also considered, and criteria for global exponential stability have been established. The paper by [28] studies the existence, uniqueness and global exponential stability of an equilibrium point for impulsive reaction–diffusion Cohen–Grossberg neural networks with time-varying delays. The authors in [29] considered impulsive reaction–diffusion Cohen–Grossberg neural networks with time-varying delays and Neumann boundary conditions. They established efficient sufficient conditions to guarantee the existence, uniqueness and global exponential stability of the equilibrium state. The global exponential stability of the equilibrium point of an impulsive reaction–diffusion delayed Cohen–Grossberg neural network has been considered in [30]. In [31], the impulsive control method is applied to a class of delayed Cohen–Grossberg neural networks with reaction–diffusion terms and Dirichlet boundary conditions. The authors proposed sufficient conditions to ensure the global exponential stability of the equilibrium point. The global exponential stability of the equilibrium point for a class of impulsive Cohen–Grossberg neural networks with time-varying delays and reaction–diffusion terms is an object of the investigation in [32]. The extensive work carried out on this class of neural networks shows their importance for theoretical avenues and practical applications.

It is clear from the cited results that the global exponential stability is an essential qualitative behavior for the class of impulsive Cohen–Grossberg neural networks with time-varying delays and reaction–diffusion terms. However, all above publications considered only single neuronal states which, in most of the cases, are equilibrium points. There are many cases when the behavior of a set of states is important. To this end, the concepts of the stability of sets [33] and manifolds [34,35] for different nonlinear problems have been introduced. Both concepts generalize the classical notion of stability of a separate state trajectory. Due to their importance in many applied models that have more than one equilibrium of interest, these concepts have been applied to some neural networks [36,37,38], including impulsive reaction–diffusion delayed Cohen–Grossberg neural network models [39,40]. In fact, there are many experiments that indicate the heterogeneity of neuronal populations growing on sets or low-dimensional manifolds [41].

However, in many practical problems, including neural network models, the stability of states depends on some constrains and restrictions. These constrains can be represented by an appropriate function *h*, which defines a specific manifold of solutions. Hence, in such cases, the concept of stability of *h*-manifolds is more appropriate, instead of considering sets or low-dimensional manifolds of solutions of a very general nature [42,43,44]. Recently, a new notion has been applied to some Cohen–Grossberg neural networks [45].

However, the important notion of the stability of *h*-manifolds is not considered for the class of impulsive reaction–diffusion Cohen–Grossberg delayed neural networks. Our aim is to contribute to the development of this direction. Also, we will extend the concept by considering the practical exponential stability case. In fact, the notions of practical stability are very applicable when a system may be unstable in the classical Lyapunov sense or *h*-manifolds sense, but its performance can be allowable from an applied point of view. Due to its importance, the concept has been applied to numerous systems [46,47,48], including neural network models [49,50,51,52]. Note that the notion of practical stability is also very useful in engineering design problems [53,54,55,56]. In addition, the exponential stability guarantees there to be a fast convergence rate for neural network models. Our goal is to study the notion of *h*-manifold practical global exponential stability in comparison with the class of impulsive reaction–diffusion Cohen–Grossberg delayed neural network models and obtain efficient criteria. These results complement some earlier publications of the *h*-manifold case [39,40] using a newly constructed Lyapunov function.

We summarize the main contributions of our paper as follows:

1. The hybrid practical stability concept with respect to *h*-manifolds is introduced to an impulsively generalized reaction–diffusion Cohen–Grossberg neural network model with time-varying delays. The introduced notion extends the concepts of impractical stability of a single state applied in [27,28,29,30,31,32] and is also well suited in applied models with several equilibrium points whose state dynamics depend on some constraints modeled by *h*-manifolds. The consideration of the notion of practical stability brings another layer of improvement as it is applicable in practical cases where ideal mathematically stable behavior is impossible.

2. The impulsive generalization of the developed model allows for the application of impulsive control strategies. Also, in contrast to the impulsive control techniques applied to similar neural network models [27,28,29,31,32], our results are not restrictive regarding the length of impulsive intervals. The upper bounds of the impulsive interval which exists in [27,28,29,31,32] are not constrained, and restrictions on the distance between impulsive moments are removed.

3. By constructing a new Lyapunov function, efficient sufficient conditions are establishes, which guarantee the practical global exponential stability with respect to a *h*-manifold. The proposed results contribute to the development of the practical stability theory for different classes of differential equations. Also, the obtained criteria are less restrictive than the stability criteria obtained in [27,28,29,30,31,32,39,40] with respect to the variable domain and diffusion coefficients.

4. A robust practical stability analysis is conducted, and criteria for robust global practical exponential stability of the introduced model under some uncertainties are proved.

5. The obtained practical global exponential stability with respect to a *h*-manifold are applied to the BAM case of the model under consideration. Thus, our results extend the results in [45] to the reaction–diffusion case.

The rest of this paper is organized according to the following scheme: In Section 2, the impulsive reaction–diffusion Cohen–Grossberg neural network model with time-varying delays is introduced. Several main definitions and lemmas on the Lyapunov direct method for impulsive reaction–diffusion delayed systems are presented. We establish our main global practical exponential stability criteria in Section 3. Due to the newly constructed Lyapunov function, the derived criteria are more relaxed with respect to the domain of the space variable and diffusion coefficients. Also, the robust practical global stability of the uncertain system is investigated. Indeed, numerous neural network models exist under the conditions of structural uncertainty due to modeling errors, measurement inaccuracy and mutations in evolutionary processes [57,58]. In addition, we apply these in Section 3 to an impulsive reaction–diffusion Cohen–Grossberg BAM neural network with time-varying delays. Some examples are presented through this Section. Finally, the conclusions are outlined in Section 4.

## 2. Impulsive Reaction–Diffusion Cohen–Grossberg Neural Networks with Time-Varying Delays: Main Notions and Definitions

Let $R^{n}$ denote the *n*-dimensional Euclidean space and $R_{+}=\left[0,\infty \right)$.

In this paper, we consider the following impulsive reaction–diffusion Cohen–Grossberg neural network model with reaction–diffusion terms and time-varying delays:

(1) $$\left\{\begin{array}{c} \frac{\partial \theta_{i}\left(t,z\right)}{\partial t}=\sum_{q=1}^{n}\frac{\partial }{\partial z_{q}}\left(D_{iq}\frac{\partial \theta_{i}\left(t,z\right)}{\partial z_{q}}\right)-a_{i}\left(\theta_{i}\left(t,z\right)\right)[b_{i}\left(\theta_{i}\left(t,z\right)\right)\\ \;-\Theta_{i}\left(t,z\right)-\sum_{j=1}^{l}v_{ij}\left(t\right)F_{j}\left(\theta_{j}\left(t,z\right)\right)\\ \;-\sum_{j=1}^{l}w_{ij}\left(t\right)G_{j}\left(\theta_{j}\left(t-\eta_{j}\left(t\right),z\right)\right)]\;\;t\neq t_{k},\\ \theta_{i}\left(t_{k}^{+},z\right)-\theta_{i}\left(t_{k},z\right)=J_{ik}\left(\theta_{i}\left(t_{k},z\right)\right), \end{array}\right.$$

where i=1,2,…,l, l≥2,k=1,2,…, t>0, $z={\left(z_{1},z_{2},\ldots ,z_{n}\right)}^{T}\in \Omega$, Ω is an open and bounded set in $R^{n}$ with a smooth boundary ∂Ω, and the measure is expressed by mesΩ>0,

In the above impulsive Cohen–Grossberg-type neural network model, $\theta_{i}\left(t,z\right)$ denotes the state of the *i*-th neural unit at time *t* (t>0) and space *z* (z∈Ω), *l* corresponds to the number of neurons in the neural network, the smooth functions $D_{iq}=D_{iq}\left(t,z\right)\geq 0$ are the diffusion coefficients along the *i*-th neuronal node, $a_{i}\left(\theta_{i}\left(t,z\right)\right)$ denotes a continuous amplification function, $b_{i}\left(\theta_{i}\left(t,z\right)\right)$ is a continuous on its domain appropriately behaved function, $v_{ij}\left(t\right)$ and $w_{ij}\left(t\right)$ are, respectively, the connection weight and time-varying delay connection weight matrices of the *j*-th neuron on the *i*-th neuron and are assumed to be continuous functions on $R_{+}$, $F_{j}\left(\theta_{j}\left(t,z\right)\right)$ and $G_{j}\left(u_{j}\left(t-\eta_{j}\left(t\right),z\right)\right)$ are the activation functions of the *j*-th neuron, $\eta_{j}\left(t\right)$ is the transmission time-varying delay and $\eta_{j}\in C\left[R,R_{+}\right],\;t>\eta_{j},\;j=1,\ldots ,l$, $0\leq \eta_{j}\left(t\right)\leq \eta ,\;\frac{d\eta_{j}\left(t\right)}{dt}<\delta_{j}$$\left(\eta >0,\;0<\delta_{j}<1\right)$, and $I_{i}\left(t,z\right)$ is the external input of the *i*-th neural unit. The points $\{t_{k}\},\;k=1,2,\ldots$ denote the moments impulsive perturbations at which abrupt changes of the state $\theta_{i}\left(t,z\right)$ from positions $\theta_{i}\left(t_{k}^{-},z\right)=\theta_{i}\left(t_{k},z\right)$ into the positions $\theta_{i}\left(t_{k}^{+},z\right)$ are observed, and $J_{ik}\left(\theta_{i}\left(t,z\right)\right)$ are impulsive functions that measure the impulsive control effects on the neuronal state $\theta_{i}\left(t,z\right)$ at the instants $t_{k}$ and space *z*. Also, we denote by $\Delta \theta_{i}\left(t_{k},z\right)=\theta_{i}\left(t_{k}^{+},z\right)-\theta_{i}\left(t_{k},z\right)$, z∈Ω, i=1,2,…,l, k=1,2,….

We assume that the sequence of discrete impulsive times $\{t_{k}\},\;k=1,2,\ldots$ is such that

$$0<t_{1}<t_{2}<\ldots <t_{k}\ldots ,\;\;\mathrm{and}\;\;t_{k}\to \infty \;\mathrm{as}\;k\to \infty .$$

**Remark** **1.** *The impulsive reaction–diffusion Cohen–Grossberg neural network model is very general and extends many existing Cohen–Grossberg neural networks* [1,2,3,4,7,8,9,10,18,19,20,21,22,51], *reaction–diffusion neural networks* [11,12,13,14,15,16,17], *impulsive cellular neural networks, impulsive Hopfield neural networks, impulsive Lotka–Volterra models* [25,52], *and some others. It provides a neural network framework for modeling real-world phenomena studied in engineering, biology, epidemiology, medicine, and finance, taking into account the advantages of (i) time-varying delays to incorporate heritable effects; (ii) reaction–diffusion terms; and (iii) the control effects of some impulsive perturbations on neural network performance.*

We will consider Model (1) under the following boundary and initial conditions:

(2) $$\theta_{i}\left(t,z\right)=0,\;t\in \left[-\eta ,\infty \right),\;z\in \partial \Omega ,$$

(3) $$\theta_{i}\left(\xi ,z\right)=\varphi_{0i}\left(\xi ,z\right),\;\xi \in \left[-\eta ,0\right],\;z\in \Omega ,$$

where $\varphi_{0}$ is the initial function, $\varphi_{0}={\left(\varphi_{01},\varphi_{02},\ldots ,\varphi_{0m}\right)}^{T}$; for any i=1,2,…,l, the functions $\varphi_{0i}\left(\xi ,z\right)$ are real-valued, defined on [−η,0]×Ω, piecewise continuous with respect to ξ with possibly finite number of discontinuity points of the first kind ξ∈[−η,0] such that $\varphi_{0i}\left(\xi^{+},z\right)$ and $\varphi_{0i}\left(\xi^{-},z\right)$ exist, and $\varphi_{0i}\left(\xi^{-},z\right)=\varphi_{0i}\left(\xi ,z\right)$, z∈Ω, i=1,2,…,l. The class of all such functions will be denoted by ${\mathrm{PC}}_{\eta }^{\Omega }$. By ${\mathrm{PCB}}_{\eta }^{\Omega }$, we denote the class of all functions $\varphi \in {\mathrm{PC}}_{\eta }^{\Omega }$ that are bounded.

The solution of the initial boundary value problem (IBVP) (1)–(3) will be denoted by

$$\theta \left(t,z\right)=\theta \left(t,z;\varphi_{0}\right).$$

**Remark** **2.** *The Dirichlet boundary conditions (2) guarantee the existence of a zero solution of Model (1). We can also use any other boundary condition that guarantees the existence of a zero solution.*

We will also denote by

- $\left||z|\right|=\sum_{q=1}^{n}\left|z_{q}\right|$—the norm of a $z={\left(z_{1},z_{2},\ldots ,z_{n}\right)}^{T}\in R^{n}$;
- ${\left||\theta \left(t,\cdot \right)|\right|}_{1}=\sum_{i=1}^{l}\int_{\Omega }\left|\theta_{i}\left(t,z\right)\right|dz$—the norm of a $\theta \left(t,z\right)={\left(\theta_{1}\left(t,z\right),\theta_{2}\left(t,z\right),\ldots ,\theta_{l}\left(t,z\right)\right)}^{T}\in R^{l}$;
- ${\left||\varphi |\right|}_{\eta }=\sup_{-\eta \leq \xi \leq 0}{\left||\varphi \left(\xi ,\cdot \right)|\right|}_{1}$—the norm of a function $\varphi \in {\mathrm{PC}}_{\eta }^{\Omega }$.

In order to apply the *h*-manifold exponential stability concept, we consider a continuous function $h=h\left(t,\theta \right),\;h:\left[-\eta ,\infty \right)\times R^{l}\to R^{p}$, l≤p, and define the following sets related to *h*:

$$\begin{array}{c} M_{t}\left(l-p\right)=\left\{\theta \in R^{l}:\;h\left(t,\theta \right)=0,\;t\in R_{+}\right\},\\ M_{t,\eta }\left(l-p\right)=\left\{\theta \in R^{l}:\;h\left(t,\theta \right)=0,\;t\in \left[-\eta ,0\right]\right\},\\ M_{t}\left(l-p\right)\left(\varepsilon \right)=\{\theta \in R^{l}{:\;||h\left(t,\theta \right)||}_{1}<\varepsilon ,\;t\in R_{+}\},\;\varepsilon >0;\\ M_{t,\eta }\left(l-p\right)\left(\varepsilon \right)=\left\{\varphi \in {\mathrm{PC}}_{\eta }^{\Omega }{:\;||h\left(\xi ,\varphi \right)||}_{\eta }<\varepsilon ,\;t\in \left[-\eta ,0\right]\right\}, \end{array}$$

where ${\left||h\left(\xi ,\varphi \right)|\right|}_{\eta }=\underset{-\eta \leq \xi \leq 0}{\sup}{\left||h\left(\xi ,\varphi \left(\xi ,\cdot \right)\right)|\right|}_{1}$.

We also assume the following assumptions to ensure the existence and uniqueness of the nodes and our findings [27,28,29,30,31,32,39,40]:

**Assumption** **1.** *The functions $a_{i}$, i=1,2,…,l, are continuous, and there exist constants ${\underline{a}}_{i}$ and ${\bar{a}}_{i}$ such that*

$$0<{\underline{a}}_{i}\leq a_{i}\left(\chi \right)\leq {\bar{a}}_{i}<\infty$$

*for χ∈R.*

**Assumption** **2.** *The functions $b_{i}$, i=1,2,…,l, are continuous, and there exist positive constants $\beta_{i}$ with*

$$\frac{b_{i}\left(\chi_{1}\right)-b_{i}\left(\chi_{2}\right)}{\chi_{1}-\chi_{2}}\geq \beta_{i}>0$$

*for $\chi_{1},\chi_{2}\in R$, $\chi_{1}\neq \chi_{2}$.*

**Assumption** **3.** *For the continuous activation functions $F_{i}$ and $G_{i}$, there exist constants $L_{i}>0$, and $M_{i}>0$ such that*

$$|F_{i}\left(\chi_{1}\right)-F_{i}\left(\chi_{2}\right)|\leq L_{i}|\chi_{1}-\chi_{2}\left|,\;\right|G_{i}\left(\chi_{1}\right)-G_{i}\left(\chi_{2}\right)|\leq M_{i}\left|\chi_{1}-\chi_{2}\right|,$$

*and $F_{i}\left(0\right)=0$, $G_{i}\left(0\right)=0$ for all $\chi_{1},\chi_{2}\in R$, $\chi_{1}\neq \chi_{2}$, i=1,2,…,l.*

**Assumption** **4.** The functions $v_{ij}$, $w_{ij}$ and $\Theta_{i}$, i,j=1,2,…,l are continuous on their domains, and there exist positive constants $\Theta_{i}^{*},\;i=1,2,\ldots ,l$ such that for $\left(t,z\right)\in R_{+}\times \Omega$, we have

$$|\Theta_{i}\left(t,z\right)|\leq \Theta_{i}^{*},\;i=1,2,\ldots ,l.$$

**Assumption** **5.** *For the diffusion coefficients $D_{iq}$, there exist constants $d_{iq}\geq 0$ such that*

$$D_{iq}\left(t,z\right)\geq d_{iq}$$

*for i=1,2,…,l, q=1,2,…,n, t>0 and z∈Ω.*

**Assumption** **6.** *The function h is continuous on $\left[-\eta ,\infty \right)\times R^{l}$, and the sets $M_{t}\left(l-p\right)$ and $M_{t,\eta }\left(l-p\right)$ are nonempty (l−p)-dimensional manifolds in $R^{l}$.*

**Assumption** **7.** *Each solution θ(t,z) of the IBVP (1)–(3) satisfying*

$${\left||h\left(t,\theta \left(t,z\right)\right)|\right|}_{1}\leq \;H<\infty$$

*is defined on $R_{+}\times \Omega$.*

The concept of *h*-manifold practical global exponential stability [42,43,44,45] will be adopted to the impulsive reaction–diffusion Cohen–Grossberg model (1) as follows:

**Definition** **1.** *The impulsive delayed reaction–diffusion Cohen–Grossberg model (1) is said to be (λ,A)-practically globally exponentially stable with respect to the function h, if given (λ,A), 0<λ<A, $\varphi_{0}\in M_{t,\eta }\left(l-p\right)\left(\lambda \right)$, which imply the existence of positive constants α, μ, such that*

$$\theta \left(t,z\right)\in M_{t}\left(l-p\right)\left(A+\alpha ||h\left(0^{+},\varphi_{0}\right){\left|\right|}_{\eta }e^{-\mu t}\right),\;t\geq 0.$$

**Remark** **3.** *The fact that System (1) is (λ,A)-practically globally exponentially stable with respect to the function h means that the h-manifold $M_{t}\left(l-p\right)$ is practically globally exponentially stable.*

**Remark** **4.** For A=0, Definition 1 is reduced to the global exponential stability of System (1) with respect to the function h, i.e., the global exponential stability of the h-manifold $M_{t}\left(l-p\right)$. The particular case of the global exponential stability of the zero solution is for h(t,θ)=θ, and the global exponential stability of any other solution of interest $\theta^{*}$ is for $h\left(t,\theta \right)=\theta -\theta^{*}$. Thus, the concept introduced by Definition 1 generalizes the exponential stability concepts studied in [28,29,30,31,32] *for similar Cohen–Grossberg models.*

In order to establish the practical global exponential stability results, we will apply the method of Lyapunov piecewise continuous functions [25].

Define $W_{k}$ as

$$W_{k}=\left\{\left(t,\theta \right):t\in \left(t_{k-1},t_{k}\right),\;\theta \in R^{l}\right\},\;k=1,2,\ldots ,\;t_{0}=0,\;\;W=\overset{\infty }{\underset{k=1}{⋃}}W_{k}.$$

**Definition** **2.** *A Lyapunov-like function $L:R_{+}\times R^{l}\to R_{+}$ belongs to the class $L_{0}$ if*

*1. L(t,θ) is continuous in W, locally Lipschitz continuous with respect to its second argument on each of the sets $W_{k}$, and L(t,0)=0 for t≥0;*

*2. For each k=1,2,… and $\theta \in R^{l}$, there exist the finite limits*

$$L\left(t_{k}^{-},\theta \right)=\lim_{\overset{t\to t_{k}}{t<t_{k}}}L\left(t,\theta \right),\;L\left(t_{k}^{+},\theta \right)=\lim_{\overset{t\to t_{k}}{t>t_{k}}}L\left(t,\theta \right),$$

*and $L\left(t_{k}^{-},\theta \right)=L\left(t_{k},\theta \right)$.*

Let $t\in R_{+}$, $t\neq t_{k}$, k=1,2,… and $\bar{\varphi }\in {\mathrm{PC}}_{\eta }^{\Omega }$. We will use the following derivative of a function $L\in L_{0}$ with respect to a system (1) given by

$$D^{+}L\left(t,\bar{\varphi }\left(0,\cdot \right)\right)=\lim_{\chi \to 0^{+}}\sup\frac{1}{\chi }\left[L(t+\chi ,\theta \left(t+\chi ,\cdot ;\bar{\varphi }\left(0,\cdot \right)\right)-L\left(t,\bar{\varphi }\left(0,\cdot \right)\right)\right].$$

The proof of the following lemma is similar to the proof of Lemma 2.1 in [51].

**Lemma** **1.** *Assume that the function $L\in L_{0}$ is such that for $t\in R_{+}$ and $\varphi \in {\mathrm{PC}}_{\eta }^{\Omega }$*

$$D^{+}L\left(t,\varphi \left(0,\cdot \right)\right)\leq -\mu L\left(t,\varphi \left(0,\cdot \right)\right)+d,\;t\neq t_{k},\;\mu ,\;d>0$$

*for*

L(t+ξ,φ(ξ,·))≤L(t,φ(0,·)),−η≤ξ≤0

*and*

$$L\left(t^{+},\varphi \left(0,\cdot \right)+\Delta \varphi \right)\leq L\left(t,\varphi \left(0,\cdot \right)\right),\;t=t_{k},\;k=1,2,\ldots .$$

*Then,*

$$L\left(t,\theta \left(t,\cdot \right)\right)\leq \underset{-\eta \leq \xi \leq 0}{\sup}L\left(0^{+},\varphi_{0}\left(\xi ,\cdot \right)\right)e^{-\mu t}+\frac{d}{\mu },\;t\geq 0.$$

**Remark** **5.** *For similar comparison results in the reaction–diffusion setting, see [39].*

**Remark** **6.** *The condition*

L(t+ξ,φ(ξ,·))≤L(t,φ(0,·)),−η≤ξ≤0

*in Lemma 1 is called the Razumikhin condition, and the corresponding technique is known as the Razumikhin technique, which is applied in* [11,25,36,39,40,45,46,48,51,52].

## 3. Practical Stability and Robust Control

### 3.1. Main Global Practical Exponential Stability Results

For a bounded continuous function *g* defined on $R_{+}$, we set

$$g^{M}=\underset{t\in R_{+}}{\sup}\left|g\left(t\right)\right|.$$

**Theorem** **1.** *Assume that Assumptions 1–7 are satisfied and*

*1. 0<λ<A are given and there exists a ν>0, such that*

$$\sum_{i=1}^{l}\Theta_{i}^{*}<A\nu .$$

*2. For the system parameters, there exists a positive number μ, such that μ>ν and $\mu =\underline{a}\mu_{1}-\bar{a}\mu_{2}$ for*

$$\mu_{1}=\min_{1\leq i\leq l}\left(\beta_{i}-\sum_{j=1}^{l}v_{ji}^{M}L_{i}\right)>0,\;\mu_{2}=\max_{1\leq i\leq l}\sum_{j=1}^{l}w_{ji}^{M}M_{i}>0,$$

*where $\underline{a}=\min_{1\leq i\leq l}{\underline{a}}_{i},\;\bar{a}=\max_{1\leq i\leq l}{\bar{a}}_{i}$.*

*3. The impulsive functions $J_{ik}$ are such that*

$$J_{ik}\left(\theta_{i}\left(t_{k},z\right)\right)=-\gamma_{ik}\theta_{i}\left(t_{k},z\right),\;\;\;\;\;0<\gamma_{ik}<2,\;and\;\left|1-\gamma_{ik}\right|\leq \frac{\underline{a}}{\bar{a}},$$

*where ${\hat{\gamma }}_{k}=\min_{1\leq i\leq l}\gamma_{ik}$, i=1,2,…,l, k=1,2,….*

*4. For the function h(t,θ), we have*

$${\left||h\left(t,\theta \right)|\right|}_{1}\leq \sum_{i=1}^{l}\int_{\Omega }\int_{0}^{\theta_{i}\left(t,z\right)}\frac{sgn(s)}{a_{i}\left(s\right)}dsdz\leq {\Lambda \left(H\right)||h\left(t,\theta \right)||}_{1},\;t\in R_{+},$$

*where Λ(H)≥1 exists for any 0<H≤∞.*

*Then, System (1) is (λ,A)-practically globally exponentially stable with respect to the function h.*

**Proof.**  Let

$$\theta \left(t,z\right)={\left(\theta_{1}\left(t,z\right),\theta_{2}\left(t,z\right),\ldots ,\theta_{l}\left(t,z\right)\right)}^{T}$$

be a solution of (1)–(3) corresponding to an initial function $\varphi_{0}\in {\mathrm{PCB}}_{\eta }^{\Omega }$, $\varphi_{0}={\left(\varphi_{01},\varphi_{02},\ldots ,\varphi_{0l}\right)}^{T}$, i.e., $\theta \left(t,z\right)=\theta \left(t,z;\varphi_{0}\right)$.

Let 0<λ<A be given and $\varphi_{0}\in M_{t,\eta }\left(l-p\right)\left(\lambda \right)$.

We define a Lyapunov function as

$$L\left(t,\theta \left(t,\cdot \right)\right)=\sum_{i=1}^{l}\int_{\Omega }\int_{0}^{\theta_{i}\left(t,z\right)}\frac{sgn(s)}{a_{i}\left(s\right)}dsdz.$$

We have from the above definition that

(4) $$\frac{1}{\bar{a}}{\left||\theta \left(t,\cdot \right)|\right|}_{1}\leq L\left(t,\theta \left(t,\cdot \right)\right)\leq \frac{1}{\underline{a}}{\left||\theta \left(t,\cdot \right)|\right|}_{1}.$$

Then, for t≥0 and $t=t_{k}$, k=1,2,…, from (4) and Condition 3 of Theorem 1, we obtain

$$L\left(t_{k}^{+},\theta \left(t_{k}^{+},\cdot \right)\right)\leq \frac{1}{\underline{a}}||\theta \left(t_{k}^{+},\cdot \right){\left|\right|}_{1}=\frac{1}{\underline{a}}||\theta \left(t_{k},\cdot \right)+\Delta \theta \left(t_{k},\cdot \right){\left|\right|}_{1}$$

$$=\frac{1}{\underline{a}}\int_{\Omega }\sum_{i=1}^{l}\left|\theta_{i}\left(t_{k},z\right)-\gamma_{ik}\theta_{i}\left(t_{k},z\right)\right|dz=\frac{1}{\underline{a}}\int_{\Omega }\sum_{i=1}^{l}\left|1-\gamma_{ik}\left|\right|\theta_{i}\left(t_{k},z\right)\right|dz$$

$$<\frac{1}{\bar{a}}\int_{\Omega }\sum_{i=1}^{l}\left|\theta_{i}\left(t_{k},z\right)\right|dz=\frac{1}{\bar{a}}||\theta \left(t_{k},\cdot \right){\left|\right|}_{1}\leq L\left(t_{k},\theta \left(t_{k},\cdot \right)\right),$$

or

(5) $$L\left(t^{+},\varphi \left(0,\cdot \right)+\Delta \varphi \right)\leq L\left(t,\varphi \left(0,\cdot \right)\right),\;t=t_{k},\;k=1,2,\ldots$$

for $\varphi \in {\mathrm{PC}}_{\eta }^{\Omega }$.

For t≥0, $t\in [t_{k-1},t_{k})$, and k=1,2,…, we have

$$\frac{d}{dt}L\left(t,\theta \left(t,\cdot \right)\right)=\sum_{i=1}^{l}\int_{\Omega }\frac{sgn(\theta_{i}\left(t,z\right))}{a_{i}\left(\theta_{i}\left(t,z\right)\right)}\frac{\partial \theta_{i}\left(t,z\right)}{\partial t}dz$$

$$=\sum_{i=1}^{l}\int_{\Omega }\frac{sgn(\theta_{i}\left(t,z\right))}{a_{i}\left(\theta_{i}\left(t,z\right)\right)}(\sum_{q=1}^{n}\frac{\partial }{\partial z_{q}}\left(D_{iq}\frac{\partial \theta_{i}\left(t,z\right)}{\partial z_{q}}\right)-a_{i}\left(\theta_{i}\left(t,z\right)\right)[b_{i}\left(\theta_{i}\left(t,z\right)\right)-\Theta_{i}\left(t,z\right)$$

$$-\sum_{j=1}^{l}v_{ij}\left(t\right)F_{j}\left(\theta_{j}\left(t,z\right)\right)-\sum_{j=1}^{l}w_{ij}\left(t\right)G_{j}\left(\theta_{j}\left(t-\eta_{j}\left(t\right),z\right)\right)])dz.$$

Without loss of generality, we can assume that, for some r=const,

$$|\theta_{i}\left(t,z\right)|\geq r\geq 0,\;t>0,\;z\in \Omega .$$

Then, from Assumption 1, we obtain

$$\int_{\Omega }\frac{sgn(\theta_{i}\left(t,z\right))}{a_{i}\left(\theta_{i}\left(t,z\right)\right)}\sum_{q=1}^{n}\frac{\partial }{\partial z_{q}}\left(D_{iq}\frac{\partial \theta_{i}\left(t,z\right)}{\partial z_{q}}\right)dz\leq \frac{1}{r\underline{a}}\int_{\Omega }\theta_{i}\left(t,z\right)\sum_{q=1}^{n}\frac{\partial }{\partial z_{q}}\left(D_{iq}\frac{\partial \theta_{i}\left(t,z\right)}{\partial z_{q}}\right)dz.$$

Now, using Assumption 5 and the zero Dirichlet boundary conditions, we have

$$\frac{1}{r\underline{a}}\sum_{q=1}^{n}\int_{\Omega }\theta_{i}\left(t,z\right)\frac{\partial }{\partial z_{q}}\left(D_{iq}\frac{\partial \theta_{i}\left(t,z\right)}{\partial z_{q}}\right)dz\leq -\frac{1}{r\underline{a}}\sum_{q=1}^{n}\int_{\Omega }d_{iq}{\left(\frac{\partial \theta_{i}\left(t,z\right)}{\partial z_{q}}\right)}^{2}dz$$

(6) $$\leq -\frac{d_{i}}{r\underline{a}}\int_{\Omega }\sum_{q=1}^{n}{\left(\frac{\partial \theta_{i}\left(t,z\right)}{\partial z_{q}}\right)}^{2}dz=-\frac{d_{i}}{r\underline{a}}\int_{\Omega }{\left|\nabla \theta_{i}\left(t,z\right)\right|}^{2}dz\leq 0,$$

where $d_{i}=\min_{1\leq q\leq n}d_{iq}$ and $\nabla \theta_{i}\left(t,z\right)={\left(\frac{\partial \theta_{i}\left(t,z\right)}{\partial z_{1}},\frac{\partial \theta_{i}\left(t,z\right)}{\partial z_{2}},\ldots ,\frac{\partial \theta_{i}\left(t,z\right)}{\partial z_{n}}\right)}^{T}$ is the gradient operator, i=1,2,…,l.

Next, from Assumptions 1–4, we obtain

$$\frac{d}{dt}L\left(t,\theta \left(t,\cdot \right)\right)\leq \sum_{i=1}^{l}\int_{\Omega }(-\beta_{i}\left|\theta_{i}\left(t,z\right)\right|$$

$$+|\Theta_{i}\left(t,z\right)|+\sum_{j=1}^{l}|v_{ij}\left(t\right)|L_{j}|\theta_{i}\left(t,z\right)|+\sum_{j=1}^{l}|w_{ij}\left(t\right)|M_{j}\left|\theta_{j}\left(t-\eta_{j}\left(t\right),z\right)\right|)dz$$

$$\leq (-\min_{1\leq i\leq l}\left(\beta_{i}-\sum_{j=1}^{l}L_{i}v_{ji}^{M})\right)\sum_{i=1}^{l}\int_{\Theta }\left|\theta_{i}\left(t,z\right)\right|dz$$

$$+\max_{1\leq i\leq l}\left(M_{i}\sum_{j=1}^{l}w_{ji}^{M}\right)\sum_{i=1}^{l}\int_{\Theta }\left|\theta_{j}\left(t-\eta_{j}\left(t\right),z\right)\right|dz)+A\nu$$

$$=-\mu_{1}{\left||\theta \left(t,\cdot \right)|\right|}_{1}+\mu_{2}\underset{t-\eta \leq \xi \leq t}{\sup}{\left||\theta \left(\xi ,\cdot \right)|\right|}_{1}+A\nu .$$

From the last estimate and (4), we obtain

(7) $$\frac{d}{dt}L\left(t,\theta \left(t,\cdot \right)\right)\leq -\underline{a}\mu_{1}L\left(t,\theta \left(t,\cdot \right)\right)+\bar{a}\mu_{2}\underset{t-\eta \leq \xi \leq t}{\sup}L\left(\xi ,\theta \left(\xi ,\cdot \right)\right)+A\nu ,\;t>0,\;t\neq t_{k}.$$

Then, (6) implies that

$$D^{+}L\left(t,\varphi \left(0,\cdot \right)\right)\leq -\left(\underline{a}\mu_{1}-\bar{a}\mu_{2}\right)L\left(t,\varphi \left(0,\cdot \right)\right)+A\nu ,\;t\neq t_{k},$$

whenever L(t+ξ,φ(ξ,·))≤L(t,φ(0,·)),−η≤ξ≤0, $\varphi \in {\mathrm{PC}}_{\eta }^{\Omega }$, t≥0.

We apply Condition 2 of Theorem 1 and obtain

(8) $$D^{+}L\left(t,\varphi \left(0,\cdot \right)\right)\leq -\mu L\left(t,\varphi \left(0,\cdot \right)\right)+A\nu ,\;t\neq t_{k},$$

whenever L(t+ξ,φ(ξ,·))≤L(t,φ(0,·)),−η≤ξ≤0, $\varphi \in {\mathrm{PC}}_{\eta }^{\Omega }$.

Then, using (5), (8) and Lemma 1, we obtain

$$L\left(t,\theta \left(t,\cdot \right)\right)\leq \underset{-\eta \leq \xi \leq 0}{\sup}L\left(0^{+},\varphi_{0}\left(\xi ,\cdot \right)\right)e^{-\mu t}+A,\;t\geq 0.$$

From Condition 4 of Theorem 1, we have

$$\left||h\left(t,\theta \left(t,z\right)\right)|\right|\leq L\left(t,\theta \left(t,\cdot \right)\right)\leq \underset{-\eta \leq \xi \leq 0}{\sup}L\left(0^{+},\varphi_{0}\left(\xi ,\cdot \right)\right)e^{-\mu t}+A$$

$$\leq \Lambda \left(H\right)||h\left(0^{+},\varphi_{0}\right){\left|\right|}_{\eta }e^{-\mu t}+A,\;t\geq 0.$$

From the last estimate, it follows that System (1) is (λ,A)-practically globally exponentially stable with respect to the function *h*, and the proof is complete. □

**Remark** **7.** *In Theorem 1, we established criteria for global practical exponential stability of the impulsive delayed reaction–diffusion Cohen–Grossberg neural network model with respect to a function h. The presented results generalize the results in* [27,28,29,30,31,32,39,40] *to the h-manifold case. For particular values of the function h, sufficient conditions can be obtained for different single-state trajectories as in* [27,28,29,30,31,32], *as well as for specific sets of trajectories as in* [39,40].

**Remark** **8.** *Theorem 1 not only generalized the results provided in* [27,28,29,30,31,32,39,40] *to the h-manifold case. The newly constructed Lyapunov-type function allows for additional advantages of the result obtained, such as (i) a very general topology of the domain* Ω* and (ii) not-heavy restrictions on the diffusion coefficients $D_{iq}$, i=1,2,…,l, q=1,2,…,n. Most of the publications considered specific sets* Ω*, such as*

$$\Omega =\{z:\;z={\left(z_{1},z_{2},\ldots ,z_{n}\right)}^{T},\;|z_{q}|<l_{q}\},$$

*where $l_{q}$(q=1,2,…,n) are positive constants (see, for example,* [39] *and some of the references cited therein), and added more restrictions on the diffusion coefficients. Thus, our results are less restrictive and can be applied in specific cases.*

**Remark** **9.** *The practical stability with respect to the h-manifold concept was investigated for impulsive Cohen–Grossberg models in* [51], *without considering the effect of reaction–diffusion terms. Hence, the proposed results extend the results in* [51] *to the reaction–diffusion case. In fact, considering reaction–diffusion terms is crucial in many applications of neural network models* [11,12,13], *including neural network models in medicine* [36,42].

**Remark** **10.** *The Lyapunov direct method is one of the most power tools applied in the study of the stability and other qualitative properties of the solutions of differential systems’ different classes. The method and related extensions are capable of establishing efficient stability criteria without knowledge about the exact solution format and have been widely applied to the stability analysis (see* [23,24,25,33,48,58], *among others), since they have played a key role in many areas such as designs and applications of neural networks. Two main approaches can be utilized when the second method of Lyapunov is applied in the study of the stability of delayed differential systems. The first one is the method of Lyapunov functions combined with the Razumikhin technique* [11,25,36,39,40,45,46,48,51,52], *which is applied in Theorem 1. It is different from the Lyapunov functional method* [30] *and leads to less restrictive criteria.*

**Remark** **11.** *Practical exponential stability behavior is very important for mathematical models, including neural networks. It includes the benefits of the notion of practical stability, which is more appropriate for applied problems when classical Lyapunov stability notions are not applicable such as engineering design tasks* [53,54,55,56]. *It also includes the benefits of exponential stability notions, which are a specific case of asymptotic stability and guarantee fast convergence rates.*

**Remark** **12.** *The proposed stability results can be successfully applied in synchronization and control problems. Thus, our results open the door for future contributions on this topic.*

**Remark** **13.** *Different control approaches, such as state-feedback control, event-triggered sampling control, and nonlinear control methods, have been applied to various neural network models* [10]. *Compared with these control schemes, we apply the impulsive control strategy, which significantly decreases the computing costs* [25,26,39,40]. *Also, in most of the existing results on impulsive control for reaction–diffusion neural networks, impulsive controllers are designed so that an upper bound for each impulsive interval* [27,28,29,31,32] *or an upper bound for the average impulsive interval* [59] *are required. Compared with the impulsive control techniques applied in* [27,28,29,31,32] *to reaction–diffusion Cohen–Grossberg neural network models, our results do not restrict the distance between impulsive moments, which greatly improves the results of recent studies.*

**Example** **1.** *Consider the impulsive delayed reaction–diffusion Cohen–Grossberg neural network model (1) for n=m=2 on the set $\Omega \subset R^{2}$, given by*

(9) $$\left\{\begin{array}{c} \frac{\partial \theta_{i}\left(t,z\right)}{\partial t}=\sum_{q=1}^{2}\frac{\partial }{\partial z_{q}}\left(D_{iq}\frac{\partial \theta_{i}\left(t,z\right)}{\partial z_{q}}\right)-a_{i}\left(\theta_{i}\left(t,z\right)\right)[b_{i}\left(\theta_{i}\left(t,z\right)\right)\\ \;-\Theta_{i}\left(t,z\right)-\sum_{j=1}^{2}v_{ij}\left(t\right)F_{j}\left(\theta_{j}\left(t,z\right)\right)\\ \;-\sum_{j=1}^{2}w_{ij}\left(t\right)G_{j}\left(\theta_{j}\left(t-\eta_{j}\left(t\right),z\right)\right)],\;t\neq t_{k},\;k=1,2,\ldots ,\\ \theta \left(t_{k}^{+},z\right)-\theta \left(t_{k},z\right)=\left(\begin{array}{cc} -2/9 & 0\\ 0 & -1/5 \end{array}\right)\theta \left(t_{k},z\right),\;k=1,2,\ldots ,\\ \theta_{i}\left(t,z\right)=0,\;t\in \left[-\eta ,\infty \right),\;z\in \partial \Omega ,\\ \theta_{i}\left(\xi ,z\right)=\varphi_{0i}\left(\xi ,z\right),\;\xi \in \left[-\eta ,0\right],\;z\in \Omega , \end{array}\right.$$

*where t>0, i=1,2, $0<t_{1}<t_{2}<\ldots <t_{k}<t_{k+1}<\ldots$, $\lim_{k\to \infty }t_{k}=\infty$, $\Theta_{1}=1$, $\Theta_{2}=\sin t$, $F_{i}\left(\theta_{i}\right)=G_{i}\left(\theta_{i}\right)=\frac{1}{2}\left(\right|\theta_{i}+1|-|\theta_{i}-1\left|\right)$, $\eta_{1}\left(t\right)=\eta_{2}\left(t\right)=e^{t}/\left(1+e^{t}\right)$, $0\leq \eta_{i}\left(t\right)\leq \eta$(η=1), $a_{i}\left(\theta_{i}\right)=1$, $b_{1}\left(\theta_{i}\right)=3\theta_{i}$, $b_{2}\left(\theta_{i}\right)=2\theta_{i}$, i=1,2,*

$${\left(v_{ij}\right)}_{2\times 2}\left(t\right)=\left(\begin{array}{cc} v_{11}\left(t\right) & v_{12}\left(t\right)\\ v_{21}\left(t\right) & v_{22}\left(t\right) \end{array}\right)=\left(\begin{array}{cc} 0.7-0.3\sin(t) & 0.2-0.3\cos(t)\\ 0.1-0.5\cos(t) & 0.1-0.2\sin(t) \end{array}\right),$$

$${\left(w_{ij}\right)}_{2\times 2}\left(t\right)=\left(\begin{array}{cc} w_{11}\left(t\right) & w_{12}\left(t\right)\\ w_{21}\left(t\right) & w_{22}\left(t\right) \end{array}\right)=\left(\begin{array}{cc} 0.3\cos(t) & 0.4\sin(t)\\ 0.4\sin(t) & 0.6\cos(t) \end{array}\right),$$

$${\left(D_{iq}\right)}_{2\times 2}=\left(\begin{array}{cc} D_{11} & D_{12}\\ D_{21} & D_{22} \end{array}\right)=\left(\begin{array}{cc} 2+\sin t & 0\\ 0 & 4+\cos\;t \end{array}\right).$$

For the particular choice of the model’s parameters, all Assumptions 1–5 are satisfied for ${\underline{a}}_{i}={\bar{a}}_{i}=\underline{a}=\bar{a}=1$, $\Theta_{i}^{*}=1$, i=1,2, $\beta_{1}=3$, $\beta_{2}=2$, $L_{1}=L_{2}=M_{1}=M_{2}=1$ and

$${\left(d_{iq}\right)}_{2\times 2}=\left(\begin{array}{cc} d_{11} & d_{12}\\ d_{21} & d_{22} \end{array}\right)=\left(\begin{array}{cc} 1 & 0\\ 0 & 3 \end{array}\right).$$

*Also, Condition 2 of Theorem 1 holds for*

$$\mu_{1}=\min_{1\leq i\leq 2}\left(\beta_{i}-\sum_{j=1}^{2}v_{ji}^{M}L_{i}\right)=1.2$$

*and*

$$\mu_{2}=\max_{1\leq i\leq 2}\sum_{j=1}^{2}w_{ji}^{M}M_{i}=1.$$

*In addition, Condition 3 of Theorem 1 is satisfied, for*

$$\gamma_{1k}=\frac{2}{9},\;\gamma_{2k}=\frac{1}{5},\;k=1,2,\ldots .$$

*We consider a continuous function $h=h\left(t,\theta \right),\;h:\left[-\eta ,\infty \right)\times R^{2}\to R$, which defines the h-manifold*

$$M_{t}\left(1\right)=\left\{\theta \in R^{2}:\;h\left(t,\theta \right)=0,\;t\in R_{+}\right\}$$

*and satisfies Condition 4 of Theorem 1.*

*Therefore, according to Theorem 1, the impulsive reaction–diffusion Cohen–Grossberg model (9) is (λ,A)-practically globally exponentially stable with respect to the function h for ν<0.2 and A>10. This means that the h-manifold $M_{t}\left(1\right)$ (which contains the zero solution, but not only) is (λ,A)-practically globally exponentially stable.*

### 3.2. Robust Global Practical Exponential Stability Results

This section will offer a robust global practical stability analysis for the impulsive reaction–diffusion Cohen–Grossberg neural network model (1). In fact, the robust control of uncertain dynamical systems is known to be of a great practical significance [57,58]. It, in general, uses strategies that guarantee an efficient performance of corresponding uncertain systems.

To this end, we will consider an impulsive reaction–diffusion Cohen–Grossberg neural network model with delays and uncertain parameters given by

(10) $$\left\{\begin{array}{c} \frac{\partial \theta_{i}\left(t,z\right)}{\partial t}=\sum_{q=1}^{n}\frac{\partial }{\partial z_{q}}\left(D_{iq}\frac{\partial \theta_{i}\left(t,z\right)}{\partial z_{q}}\right)-a_{i}\left(\theta_{i}\left(t,z\right)\right)[(b_{i}\left(\theta_{i}\left(t,z\right)\right))\\ \;-\left(\Theta_{i}\left(t,z\right)+{\tilde{\Theta }}_{i}\left(t,z\right)\right)-\sum_{j=1}^{l}\left(v_{ij}\left(t\right)+{\tilde{v}}_{ij}\left(t\right)\right)F_{j}\left(\theta_{j}\left(t,z\right)\right)\\ \;-\sum_{j=1}^{l}\left(w_{ij}\left(t\right)+{\tilde{w}}_{ij}\left(t\right)\right)(G_{j}\left(\theta_{j}\left(t-\eta_{j}\left(t\right),z\right)\right))],\;t\neq t_{k},\\ \theta_{i}\left(t_{k}^{+},z\right)-\theta_{i}\left(t_{k},z\right)=-\left(\gamma_{ik}+{\tilde{\gamma }}_{ik}\right)\theta_{i}\left(t_{k},z\right), \end{array}\right.$$

where t>0, z∈Ω, and ${\tilde{v}}_{ij},{\tilde{w}}_{ij},{\tilde{\Theta }}_{i}$ are all continuous functions in their domains, and the constants ${\tilde{\gamma }}_{ik}$ represent the uncertain terms in the impulsive functions for i,j=1,…,l,k=1,2,…. The original system (1) is called a ”nominal system” of Model (10) when all uncertain parameters are zeros [57].

**Definition** **3.** *The impulsive reaction–diffusion Cohen–Grossberg model (1) is called (λ,A)-practically globally robustly exponentially stable with respect to the function h, if, for a given (λ,A) with 0<λ<A, $\varphi_{0}\in M_{t,\eta }\left(l-p\right)\left(\lambda \right)$, System (10) is (λ,A)-practically globally exponentially stable with respect to the function h for any ${\tilde{v}}_{ij}$, ${\tilde{w}}_{ij}$, ${\tilde{\Theta }}_{i}$ and ${\tilde{\gamma }}_{ik}$, i,j=1,…,l, k=1,2,….*

Now, we need the following assumptions:

**Assumption** **8.** *The unknown functions ${\tilde{v}}_{ij},\;{\tilde{w}}_{ij}$ and ${\tilde{\Theta }}_{i}$ are bounded on their domains, and*

$$\left|\right|{\tilde{\Theta }}_{i}\left(t,z\right)\left|\right|\leq {\tilde{\Theta }}_{i}^{*},\;\left(t,z\right)\in R_{+}\times \Omega ,$$

where ${\tilde{\Theta }}_{i}^{*}>0$, i,j=1,2,…,l.

**Assumption** **9.** *The unknown constants ${\tilde{\gamma }}_{ik}$ are such that $0<{\tilde{\gamma }}_{ik}<2-\gamma_{ik},\;i=1,2,\ldots ,l,\;k=1,2,\ldots$.*

The proof of the next theorem follows directly from Theorem 1.

**Theorem** **2.** *Assume that Assumpstion 1–9 hold*, *and that*

*1. Conditions 1 and 5 of the theorem hold.*

2. 0<λ<A are given, and there exists a $\nu^{*}>0$ such that

$$\sum_{i=1}^{l}\left(\Theta_{i}^{*}+{\tilde{\Theta }}_{i}^{*}\right)<A\nu^{*}.$$

3. There exists a positive number $\mu^{*}$ such that $\mu^{*}>\nu^{*}$ and $\mu^{*}=\underline{a}\mu_{1}^{*}-\bar{a}\mu_{2}^{*}$ for

$$\mu_{1}^{*}=\min_{1\leq i\leq m}\left(\beta_{i}-\sum_{j\neq i}^{l}\left(v_{ji}^{M}+{\tilde{v}}_{ji}^{M}\right)L_{i}\right)>0,$$

$$\mu_{2}^{*}=\max_{1\leq i\leq l}\sum_{j=1}^{l}\left(w_{ij}^{M}+{\tilde{w}}_{ij}^{M}\right)Mi>0.$$

*Then, System (1) is (λ,A)-practically robustly globally robustly exponentially stable with respect to the function h.*

**Example** **2.** *Consider the following 2D uncertain impulsive reaction–diffusion Cohen–Grossberg neural network model:*

(11) $$\left\{\begin{array}{c} \frac{\partial \theta_{i}\left(t,z\right)}{\partial t}=\sum_{q=1}^{n}\frac{\partial }{\partial z_{q}}\left(D_{iq}\frac{\partial \theta_{i}\left(t,z\right)}{\partial z_{q}}\right)-a_{i}\left(\theta_{i}\left(t,z\right)\right)[(b_{i}\left(\theta_{i}\left(t,z\right)\right))\\ \;-\left(\Theta_{i}\left(t,z\right)+{\tilde{\Theta }}_{i}\left(t,z\right)\right)-\sum_{j=1}^{l}\left(v_{ij}\left(t\right)+{\tilde{v}}_{ij}\left(t\right)\right)F_{j}\left(\theta_{j}\left(t,z\right)\right)\\ \;-\sum_{j=1}^{l}\left(w_{ij}\left(t\right)+{\tilde{w}}_{ij}\left(t\right)\right)(G_{j}\left(\theta_{j}\left(t-\eta_{j}\left(t\right),z\right)\right))],\;t\neq t_{k},\\ \Delta \theta \left(t_{k}^{+},z\right)=\left(\begin{array}{cc} -\frac{2}{9}-{\tilde{\gamma }}_{1k} & 0\\ 0 & -\frac{1}{5}-{\tilde{\gamma }}_{2k} \end{array}\right)\theta \left(t_{k},z\right),\;k=1,2,\ldots , \end{array}\right.$$

*where i=1,2, t>0, for which System (9) is the nominal system, and the continuous $R_{+}$ functions ${\tilde{v}}_{ij},\;{\tilde{w}}_{ij}$, the continuous $R_{+}\times \Omega$ function ${\tilde{\Theta }}_{i}$, i,j=1,2,k=1,2,…, and constants ${\tilde{\gamma }}_{ik},\;i=1,2,\;k=1,2,\ldots$ are the uncertain parameters.*

*If uncertain terms are bounded and all conditions of Theorem 2 are satisfied, System (9) is (λ,A)-practically globally robustly exponentially stable with respect to the function h, which determines the h-manifold $M_{t}\left(1\right)$.*

### 3.3. The BAM Case

The newly established global practical exponential stability results in Theorem 1 can be applied to various classes of impulsive reaction–diffusion Cohen–Grossberg delayed neural network models. One of the important classes of such models is a specific class of BAM models, introduced initially in [60,61].

We will apply the obtained criteria for the global practical stability to the following system of BAM neural networks:

(12) $$\left\{\begin{array}{c} \frac{\partial \theta_{i}\left(t,z\right)}{\partial t}=\sum_{q=1}^{n}\frac{\partial }{\partial z_{q}}\left(D_{iq}\frac{\partial \theta_{i}\left(t,z\right)}{\partial z_{q}}\right)-a_{i}\left(\theta_{i}\left(t,z\right)\right)[b_{i}\left(\theta_{i}\left(t,z\right)\right)\\ \;-\sum_{j=1}^{m}v_{ji}\left(t\right){\hat{F}}_{j}\left(\zeta_{j}\left(t,z\right)\right)-\sum_{j=1}^{m}w_{ji}\left(t\right){\hat{G}}_{j}\left(\zeta_{j}\left(t-{\hat{\eta }}_{j}\left(t\right),z\right)\right)-\Theta_{i}\left(t,z\right)],\\ \frac{\partial \zeta_{j}\left(t,z\right)}{\partial t}=\sum_{q=1}^{n}\frac{\partial }{\partial z_{q}}\left({\hat{D}}_{jq}\frac{\partial \zeta_{j}\left(t,z\right)}{\partial z_{q}}\right)-{\hat{a}}_{j}\left(\zeta_{j}\left(t,z\right)\right)[{\hat{b}}_{j}\left(\zeta_{j}\left(t,z\right)\right)\\ \;-\sum_{i=1}^{l}p_{ij}\left(t\right)F_{i}\left(\theta_{i}\left(t,z\right)\right)-\sum_{i=1}^{l}q_{ij}\left(t\right)G_{i}\left(\theta_{i}\left(t-\eta_{i}\left(t\right),z\right)\right)-{\hat{\Theta }}_{j}\left(t,z\right)],\;\;\;t\neq t_{k},\\ \theta_{i}\left(t_{k}^{+},z\right)-\theta_{i}\left(t_{k},z\right)=-\gamma_{ik}\theta_{i}\left(t_{k},z\right),\\ \zeta_{j}\left(t_{k}^{+},z\right)-\zeta_{j}\left(t_{k},z\right)=-\sigma_{jk}\zeta_{j}\left(t_{k},z\right), \end{array}\right.$$

where i=1,2,…,l,j=1,2,…,m, the model parameter functions $D_{iq}=D_{iq}\left(t,z\right)$ and ${\hat{D}}_{jq}={\hat{D}}_{jq}\left(t,z\right)$ are positive and continuous on $R_{+}\times \Omega$, $a_{i},{\hat{a}}_{j},b_{i},{\hat{b}}_{j}$, $Fj,{\hat{F}}_{i},G_{j},{\hat{G}}_{i}$$v_{ji},w_{ji},p_{ij},q_{ij}\in C\left[R,R\right]$, $\eta_{j},{\hat{\eta }}_{i}\in C\left[R,R_{+}\right]$, $t>\eta_{j}$, $t>{\hat{\eta }}_{i}$, $0\leq \eta_{j}\left(t\right)\leq \eta$, $\frac{d\eta_{j}\left(t\right)}{dt}<\delta_{j}$$\left(\delta_{j}<1\right)$, $0\leq {\hat{\eta }}_{i}\left(t\right)\leq \hat{\eta },\;\frac{d{\hat{\eta }}_{i}\left(t\right)}{dt}<{\hat{\delta }}_{i}$$\left({\hat{\delta }}_{i}<1\right)$, $\Theta_{i},{\hat{\Theta }}_{j}\in C\left[R\times \Omega ,R\right]$, $\gamma_{ik},\sigma_{jk}\in R,\;k=1,2,\ldots$, and the impulsive times $\{t_{k}\},\;k=1,2,\ldots$ are such that

$$0<t_{1}<t_{2}<\ldots <t_{k}\ldots ,\;\;\mathrm{and}\;\;t_{k}\to \infty \;\mathrm{as}\;k\to \infty .$$

Denote by $\tau =\max\{\eta ,\hat{\eta }\}$. We will consider the following boundary and initial conditions associated with Model (12):

(13) $$\theta_{i}\left(t,z\right)=0,\;\zeta_{j}\left(t,z\right)=0,\;t\in \left[-\tau ,\infty \right),\;x\in \partial \Omega ,$$

(14) $$\theta_{i}\left(\xi ,z\right)=\varphi_{0i}\left(\xi ,z\right),\;\zeta_{j}\left(\xi ,z\right)=\psi_{0j}\left(\xi ,z\right),\;\xi \in \left[-\tau ,0\right],\;z\in \Omega ,$$

where $\varphi_{0i}\left(\xi ,z\right)$$\psi_{0j}\left(\xi ,z\right)$, i=1,2,…,l, j=1,2,…,m, are real–valued functions that are well defined on [−τ,0]×Ω, piecewise continuous with respect to ξ with a possibly finite number of discontinuity points of the first kind ξ∈[−τ,0], and the functions $\phi_{0}={\left(\varphi_{0},\psi_{0}\right)}^{T}$, $\varphi_{0}={\left(\varphi_{01},\varphi_{02},\ldots ,\varphi_{0l}\right)}^{T}$, and $\psi_{0}={\left(\psi_{01},\psi_{02},\ldots ,\psi_{0m}\right)}^{T}$ belong to the class ${\mathrm{PCB}}_{\tau }^{\Omega }$.

We will again consider the following norms:

$${\left||u\left(t,\cdot \right)|\right|}_{1}=\sum_{i=1}^{l}\int_{\Omega }\left|\theta_{i}\left(t,z\right)\right|dz+\sum_{j=1}^{m}\int_{\Omega }\left|\zeta_{j}\left(t,z\right)\right|dz$$

of a $u={\left(\theta ,\zeta \right)}^{T}\in R^{l+m}$,

$$u=u\left(t,z\right)={\left(\theta \left(t,z\right),\zeta \left(t,z\right)\right)}^{T}={\left(\theta_{1}\left(t,z\right),\theta_{2}\left(t,z\right),\ldots ,\theta_{l}\left(t,z\right),\zeta_{1}\left(t,z\right),\zeta_{2}\left(t,z\right),\ldots ,\zeta_{m}\left(t,z\right)\right)}^{T}$$

and

$${\left||\phi |\right|}_{\tau }=\underset{-\tau \leq \xi \leq 0}{\sup}{\left||\phi \left(\xi ,\cdot \right)|\right|}_{1}$$

for a $\phi \in {\mathrm{PC}}_{\tau }^{\Omega }$.

In order to apply the *h*-manifolds concept and Theorem 1, we consider a continuous function $h:\left[-\tau ,\infty \right)\times R^{l+m}\to R^{p}$, the corresponding *h*-manifolds $M_{t}\left(l+m-p\right)$, $M_{t,\tau }\left(l+m-p\right)$, $M_{t}\left(l+m-p\right)\left(\varepsilon \right)$ and $M_{t,\tau }\left(l+m-p\right)\left(\varepsilon \right)$, and we assume that $M_{t}\left(l+m-p\right)$ and $M_{t,\tau }\left(l+m-p\right)$ are nonempty (l−p)-dimensional manifolds in $R^{l+m}$.

By using the above *h*-manifolds, Definition 1 will be modified as follows:

**Definition** **4.** *The impulsive delayed reaction–diffusion BAM Cohen–Grossberg model (12) is said to be (λ,A)-practically globally exponentially stable with respect to the function h, if given (λ,A), 0<λ<A, with $\phi_{0}\in M_{t,\tau }\left(l+m-p\right)\left(\lambda \right)$ implying the existence of positive constants α, μ, such that*

$$u\left(t,z\right)\in M_{t}\left(l+m-p\right)\left(A+\alpha ||h\left(0^{+},\phi_{0}\right){\left|\right|}_{\tau }e^{-\mu t}\right),\;t\geq 0.$$

We introduce the following conditions:

**Assumption** **10.** *The functions ${\hat{a}}_{j}$, j=1,2,…,m, are continuous, and there exist constants ${\underline{\hat{a}}}_{j}$ and ${\bar{\hat{a}}}_{j}$ such that*

$$0<{\underline{\hat{a}}}_{j}\leq {\hat{a}}_{j}\left(\chi \right)\leq {\bar{\hat{a}}}_{j}<\infty$$

*for χ∈R.*

**Assumption** **11.** *The functions ${\hat{b}}_{j}$, j=1,2,…,m, are continuous, and there exist positive constants ${\hat{\beta }}_{j}$ with*

$$\frac{{\hat{b}}_{j}\left(\chi_{1}\right)-{\hat{b}}_{j}\left(\chi_{2}\right)}{\chi_{1}-\chi_{2}}\geq {\hat{\beta }}_{j}>0$$

*for $\chi_{1},\chi_{2}\in R$, $\chi_{1}\neq \chi_{2}$.*

**Assumption** **12.** *For the continuous activation functions ${\hat{F}}_{j}$ and ${\hat{G}}_{j}$, there exist constants ${\hat{L}}_{j}>0$ and ${\hat{M}}_{j}>0$ such that*

$$|{\hat{F}}_{j}\left(\chi_{1}\right)-{\hat{F}}_{j}\left(\chi_{2}\right)|\leq {\hat{L}}_{j}|\chi_{1}-\chi_{2}\left|,\;\right|{\hat{G}}_{j}\left(\chi_{1}\right)-{\hat{G}}_{j}\left(\chi_{2}\right)|\leq {\hat{M}}_{j}\left|\chi_{1}-\chi_{2}\right|,$$

*and ${\hat{F}}_{j}\left(0\right)=0$, ${\hat{G}}_{j}\left(0\right)=0$ for all $\chi_{1},\chi_{2}\in R$, $\chi_{1}\neq \chi_{2}$, j=1,2,…,m.*

**Assumption** **13.** The functions $p_{ij}$, $q_{ij}$ and ${\hat{\Theta }}_{j}$, i=1,2,…,l, j=1,2,…,m, are continuous on their domains, and there exist positive constants ${\hat{\Theta }}_{i}^{*},\;i=1,2,\ldots ,l$ such that for $\left(t,z\right)\in R_{+}\times \Omega$:

$$|{\hat{\Theta }}_{j}\left(t,z\right)|\leq {\hat{\Theta }}_{j}^{*},\;j=1,2,\ldots ,m.$$

**Assumption** **14.** *For the diffusion coefficients ${\hat{D}}_{jq}$, there exist constants ${\hat{d}}_{jq}\geq 0$ such that*

$${\hat{D}}_{jq}\left(t,z\right)\geq {\hat{d}}_{jq}$$

*for j=1,2,…,m, q=1,2,…,n, t>0 and z∈Ω.*

**Assumption** **15.** *Each solution u(t,z) of the IBVP (12)–(14) satisfying*

$${\left||h\left(t,u\left(t,z\right)\right)|\right|}_{1}\leq \;\hat{H}<\infty$$

*is defined on $R_{+}\times \Omega$.*

By using a function $L:R_{+}\times R^{l+m}\to R_{+}$ from the class $L_{0}$ defined by

$$L\left(t,u\left(t,\cdot \right)\right)=\sum_{i=1}^{l}\int_{\Omega }\int_{0}^{\theta_{i}\left(t,z\right)}\frac{sgn(s)}{a_{i}\left(s\right)}dsdz+\sum_{j=1}^{m}\int_{\Omega }\int_{0}^{\zeta_{j}\left(t,z\right)}\frac{sgn(s)}{{\hat{a}}_{j}\left(s\right)}dsdz,$$

the next result follows directly from Theorem 1.

**Theorem** **3.** *Assume that Assumptions 1–7 and 10–15 are satisfied. Moreover, assume the following:*

*1. 0<λ<A are given, and there exists a ν>0 such that*

$$\sum_{i=1}^{l}\Theta_{i}^{*}+\sum_{j=1}^{m}{\hat{\Theta }}_{i}^{*}<A\hat{\nu }.$$

*2. There exists a positive number $\hat{\mu }$, $\hat{\mu }>\hat{\nu }$, and $\hat{\mu }={\hat{\mu }}_{1}-{\hat{\mu }}_{2}$, where*

$${\hat{\mu }}_{1}=\left(\min_{1\leq i\leq l}\left({\underline{a}}_{i}\beta_{i}-{\bar{a}}_{i}\sum_{j=1}^{m}p_{ij}^{M}L_{i}\right)+\min_{1\leq j\leq m}\left({\underline{\hat{a}}}_{j}{\hat{\beta }}_{j}-{\bar{\hat{a}}}_{j}\sum_{i=1}^{l}v_{ji}^{M}{\hat{L}}_{j}\right)\right)$$

$$>{\hat{\mu }}_{2}=\max_{1\leq j\leq m}{\bar{\hat{a}}}_{j}\sum_{i=1}^{m}w_{ij}^{M}{\hat{M}}_{j}+{\bar{a}}_{i}\max_{1\leq i\leq l}\sum_{j=1}^{l}q_{ji}^{M}M_{i}.$$

*3. The constants $\gamma_{ik}$ and $\sigma_{jk}$ are such that*

$$0<\gamma_{ik}<2,\;0<\sigma_{jk}<2,$$

*and*

$$\max\left(|1-{\hat{\gamma }}_{k}|,|1-{\hat{\sigma }}_{k}|\right)\leq \frac{\underline{a}}{\bar{a}},$$

*where ${\hat{\gamma }}_{k}=\min_{1\leq i\leq l}\gamma_{ik},\;{\hat{\sigma }}_{k}=\min_{1\leq j\leq m}\sigma_{jk},\;k=1,2,\ldots$, and*

$$\underline{a}=\min\left(\min_{1\leq i\leq l}{\underline{a}}_{i},\min_{1\leq j\leq m}{\underline{\hat{a}}}_{j}\right),$$

$$\bar{a}=\max\left(\max_{1\leq i\leq l}{\bar{a}}_{i},\max_{1\leq j\leq m}{\bar{\hat{a}}}_{j}\right).$$

*4. For the function h(t,u), we have*

$${\left||h\left(t,u\right)|\right|}_{1}\leq L\left(t,u\left(t,\cdot \right)\right)\leq \Lambda \left(\hat{H}\right)||h\left(t,u\right){\left|\right|}_{1},\;t\in R_{+},$$

*where $\Lambda (\hat{H})\geq 1$ exists for any $0<\hat{H}\leq \infty$.*

*Then, System (12) is (λ,A)-practically globally exponentially stable with respect to the function h.*

**Example** **3.** *Consider the following impulsive reaction–diffusion Cohen–Grossberg-type BAM neural networks with time-varying delays:*

(15) $$\left\{\begin{array}{c} \frac{\partial \theta_{i}\left(t,z\right)}{\partial t}=\frac{\partial }{\partial z}\left(D_{i}\frac{\partial \theta_{i}\left(t,z\right)}{\partial z}\right)-a_{i}\left(\theta_{i}\left(t,z\right)\right)[b_{i}\left(\theta_{i}\left(t,z\right)\right)\\ \;-\sum_{j=1}^{2}v_{ji}\left(t\right){\hat{F}}_{j}\left(\zeta_{j}\left(t,z\right)\right)-\sum_{j=1}^{2}w_{ji}\left(t\right){\hat{G}}_{j}\left(\zeta_{j}\left(t-{\hat{\eta }}_{j}\left(t\right),z\right)\right)-\Theta_{i}\left(t,z\right)],\\ \frac{\partial \zeta_{j}\left(t,z\right)}{\partial t}=\frac{\partial }{\partial z}\left({\hat{D}}_{j}\frac{\partial \zeta_{j}\left(t,z\right)}{\partial z}\right)-{\hat{a}}_{j}\left(\zeta_{j}\left(t,z\right)\right)[{\hat{b}}_{j}\left(\zeta_{j}\left(t,z\right)\right)\\ \;-\sum_{i=1}^{2}p_{ij}\left(t\right)F_{i}\left(\theta_{i}\left(t,z\right)\right)-\sum_{i=1}^{2}q_{ij}\left(t\right)G_{i}\left(\theta_{i}\left(t-\eta_{i}\left(t\right),z\right)\right)-{\hat{\Theta }}_{j}\left(t,z\right)],\;\;\;t\neq t_{k}, \end{array}\right.$$

*with impulsive perturbations of the type*

(16) $$\begin{array}{c} \theta \left(t_{k}^{+},z\right)-\theta \left(t_{k},z\right)=\left(\begin{array}{cc} -1+\frac{1}{3k} & 0\\ 0 & -1+\frac{1}{3k} \end{array}\right)\theta \left(t_{k},z\right),\;k=1,2,\ldots ,\\ \sigma {(}_{k}t^{+},z)-\sigma \left(t_{k},z\right)=\left(\begin{array}{cc} -1+\frac{1}{2k} & 0\\ 0 & -1+\frac{1}{2k} \end{array}\right)\sigma \left(t_{k},z\right),\;k=1,2,\ldots ,\;z\in \Omega \end{array}$$

*where t>0, Ω=(−1,1),*

$$\theta \left(t,z\right)=\left(\begin{array}{c} \theta_{1}\left(t,z\right)\\ \theta_{2}\left(t,z\right) \end{array}\right),\;\zeta \left(t,z\right)=\left(\begin{array}{c} \zeta_{1}\left(t,z\right)\\ \zeta_{2}\left(t,z\right) \end{array}\right),\;\Theta_{1}=\Theta_{2}={\hat{\Theta }}_{1}={\hat{\Theta }}_{2}=1,$$

$$F_{i}\left(\theta_{i}\right)=G_{i}\left(\theta_{i}\right)=\frac{|\theta_{i}+1|-|\theta_{1}-1|}{2},\;{\hat{F}}_{j}\left(\zeta_{j}\right)={\hat{G}}_{j}\left(\zeta_{j}\right)=\frac{|\zeta_{j}+1|-|\zeta_{j}-1|}{2},\;i,j=1,2$$

$$0\leq \eta_{i}\left(t\right)\leq 1,\;0\leq {\hat{\eta }}_{j}\left(t\right)\leq 1,\;a_{i}\left(\theta_{i}\right)={\hat{a}}_{j}\left(\zeta_{j}\right)=1,\;b_{1}\left(\theta_{i}\right)=2\theta_{i},\;b_{2}\left(\theta_{i}\right)=3\theta_{i},$$

$${\hat{b}}_{1}\left(\zeta_{j}\right)={\hat{b}}_{2}\left(\zeta_{j}\right)=2\zeta_{j},\;i,j=1,2,$$

$${\left(v_{ij}\right)}_{2\times 2}=\left(\begin{array}{cc} v_{11} & v_{12}\\ v_{21} & v_{22} \end{array}\right)=\left(\begin{array}{cc} 1 & 0.5\\ 0.6 & -0.5 \end{array}\right),\;{\left(w_{ij}\right)}_{2\times 2}=\left(\begin{array}{cc} w_{11} & w_{12}\\ w_{21} & w_{22} \end{array}\right)=\left(\begin{array}{cc} 0.3 & 0.4\\ -0.4 & 0.2 \end{array}\right),$$

$${\left(p_{ij}\right)}_{2\times 2}=\left(\begin{array}{cc} p_{11} & p_{12}\\ p_{21} & p_{22} \end{array}\right)=\left(\begin{array}{cc} 0.7 & -0.6\\ 0.9 & 0.8 \end{array}\right),\;{\left(q_{ij}\right)}_{2\times 2}=\left(\begin{array}{cc} q_{11} & q_{12}\\ q_{21} & q_{22} \end{array}\right)=\left(\begin{array}{cc} 0.2 & -0.1\\ 0.1 & -0.2 \end{array}\right),$$

$${\left(D_{iq}\right)}_{2\times 2}=\left(\begin{array}{cc} D_{11} & D_{12}\\ D_{21} & D_{22} \end{array}\right)=\left(\begin{array}{cc} 1 & 0\\ 0 & 3 \end{array}\right),$$

$${\left({\hat{D}}_{jq}\right)}_{2\times 2}=\left(\begin{array}{cc} D_{11} & D_{12}\\ D_{21} & D_{22} \end{array}\right)=\left(\begin{array}{cc} 2 & 0\\ 0 & 3 \end{array}\right),$$

$$0<t_{1}<t_{2}<\ldots <t_{k}<t_{k+1}<\ldots ,\;\lim_{k\to \infty }t_{k}=\infty .$$

*We have that all assumptions of Theorem 3 are satisfied for*

$$\begin{array}{c} L_{1}=L_{2}=1,\;M_{1}=M_{2}=1,\;{\hat{L}}_{1}={\hat{L}}_{2}=1,\;{\hat{M}}_{1}={\hat{M}}_{2}=1,\\ {\underline{a}}_{i}={\bar{a}}_{i}=1,\;\hat{{\underline{a}}_{j}}=\hat{{\bar{a}}_{j}}=1,\;\beta_{1}=2,\;\beta_{2}=3,\;{\hat{\beta }}_{1}={\hat{\beta }}_{2}=2. \end{array}$$

*More precisely, Condition 2 of Theorem 3 is satisfied for $0<\hat{\mu }\leq 0.2$.*

*In addition*, *Condition 3 of Theorem 3 is true since $\gamma_{ik}=1-\frac{1}{3k}$, $\sigma_{jk}=1-\frac{1}{2k}$ for i,j=1,2, k=1,2,… and*

$$\frac{1}{2}=\max\left(|1-{\hat{\gamma }}_{k}|,|1-{\hat{\sigma }}_{k}|\right)\leq \frac{\underline{a}}{\bar{a}}=1.$$

*We consider a continuous function $h=h\left(t,\theta ,\zeta \right)=h\left(t,u\right),\;h:\left[-\tau ,\infty \right)\times R^{4}\to R$, which defines the h-manifold*

$$M_{t}\left(3\right)=\left\{u\in R^{4}:\;h\left(t,u\right)=0,\;t\in R_{+}\right\}$$

*and satisfies Condition 4 of Theorem 3.*

*Therefore, according to Theorem 3, the impulsive reaction–diffusion Cohen–Grossberg models (15) and (16) are (λ,A)-practically globally exponentially stable with respect to the function h for $\hat{\nu }<0.2$ and A>20.*

**Remark** **14.** *Theorem 3 generalizes Theorem 1 to the BAM case. Also, it extends similar results for impulsive Cohen–Grossberg BAM neural networks* [45] *to the reaction–diffusion case. Again, the consideration of a new Lyapunov function leads to less restrictive criteria with respect to the domain* Ω *and the diffusion coefficients. Also, Example 3 demonstrates the efficiency of the obtained criteria.*

## 4. Conclusions

In this paper, a class of impulsive reaction–diffusion Cohen–Grossberg neural networks with time-varying delays was investigated. The concept of practically globally exponentially stable *h*-manifolds was applied to the proposed model, and the corresponding stability analysis was performed. Efficient sufficient conditions were established using a newly constructed Lyapunov-type function. With this research, we extend the single-state global exponential stability results on such neural network models to the *h*-manifold case. Also, the obtained criteria are less restrictive than similar ones in the existing literature with respect to the domain of the systems’ variable, diffusion coefficients and impulsive intervals. In addition, the effect of some uncertain parameters is studied. The BAM neural network model with impulsive perturbations, reaction–diffusion terms and time-varying delays is also considered. Moreover, *h*-manifolds’ practical global exponential stability results are applied to it. The presented results can be extended to complex systems with reaction–diffusion terms. Future directions of our research will be aimed at stochastic neural network models and systems with fractional-order dynamics.

## Data Availability

The original contributions presented in this study are included in the article. Further inquiries can be directed to the corresponding author.
